# Chemical Preservatives in Food

**Published:** 1899-12-02

**Authors:** 


### CHEMICAL PRESERVATIVES IN FOOD.

After a very full investigation of the subject of the influence upon health of chemical preservatives contained in food, Dr. Foulerton,[1] bacteriologist to the Middlesex Hospital, says that he is of opinion that the amount of preservative, the same not being injurious to health, which might under ordinary circumstances be permitted in milk, should be limited to such a quantity as will suffice to keep the milk sweet and fit for use as human food for a period of 24 hours in the hottest weather. This amount he fixes at about the following quantities: Of boric acid or boric mixture (boric acid and sodium bi-borate) 35 grains per gallon, or 1 part in 2,000; and of formic aldehyde 1 part in 50,000, or 1 part of formalin in 20,000. As to the effect of such quantities upon the health, he says that the amount of boric acid ordinarily taken in milk by the healthy adult (assuming, of course, that no other similarly preserved article of food were taken) must be regarded as absolutely negligable. It would seem, however, that the matter is otherwise in regard to an invalid on milk diet, for certain cases have been published which show that the amount taken as a preservative would with some patients have a decidedly injurious effect. "These cases (and others, if necessary, might be quoted) clearly show that the doses of boric acid about equivalent to the amount which an invalid adult on milk diet might take, if the preservative were present in the proportion of 1 part per 2,000, are capable of producing serious toxic symptoms. And it must be assumed that the proportionately greater amount taken by the infant in preserved milk might be no less injurious." There one may be well content to leave the matter. It is adults, not infants, who make our laws, and, however great the selfishness of man may be, we doubt if healthy adults will be found willing to make laws which, while harmless to themselves, will lead to the poisoning of infants and of the sick.

[1] Lancet, Nov. 25.

